# Geosocial Networking Dating App Usage and Risky Sexual Behavior in Young Adults Attending a Music Festival: Cross-sectional Questionnaire Study

**DOI:** 10.2196/21082

**Published:** 2021-04-15

**Authors:** Shirali Garga, Meryl Thomas, Ashneet Bhatia, Aidan Sullivan, Franklin John-Leader, Sabrina Pit

**Affiliations:** 1 University Centre for Rural Health Western Sydney University Lismore Australia; 2 Harm Reduction and Health Promotion Programs, HIV and Related Programs (HARP), North Coast Public Health Mid-North Coast Local Health District Lismore Australia; 3 University Centre for Rural Health University of Sydney Lismore Australia; 4 NSW Rural Doctors Network Newcastle Australia

**Keywords:** sexual health, mobile apps, young adults, music festival

## Abstract

**Background:**

Despite the prevalent use of geosocial networking dating apps (GNDAs), there is limited research on their impact on sexual health outcomes among young music festivals attendees.

**Objective:**

This study aims to explore the use of GNDAs and risky sexual behaviors of young adults attending a music festival.

**Methods:**

The music festival attendees (N=862) completed a cross-sectional questionnaire study encompassing demographics, dating app use, and risky sexual behaviors in the past year. Associations between these variables were estimated using bivariate and multivariate logistic regression analyses.

**Results:**

Of the respondents, 51.9% (448/862) had used GNDAs in the previous year. Compared with people who had 1 partner, people who had 2-5 sexual partners in the previous year had almost 7 times the odds of using dating apps (odds ratio [OR] 6.581, 95% CI 4.643-9.328) and those who had more than 5 partners had 14 times the odds of using dating apps (OR 14.294, 95% CI 8.92-22.906). Condom users were more likely to be app users (*P*<.001), as were those who relied on emergency Plan B (*P*=.002), but people using hormonal contraception were less likely to use dating apps (*P*=.004). After adjusting for sexual orientation and relationship status, those having casual sex had 3.096 (95% CI 2.225-4.307; *P*<.001) times the odds of using dating apps and those having multiple sexual partners had 3.943 (95% CI 2.782-5.588; *P*<.001) times the odds of using dating apps. Similarly, after adjusting for sexual orientation, relationship status, and number of sexual partners, people who had no discussions before having sex about sexually transmitted infections (STIs) or boundaries were more likely to use dating apps (OR 1.755, 95% CI 1.232-2.500; *P*=.002). Those who perceived the risk of having sex without contraception to be *very high* had 2.486 (95% CI 2.213-5.096; *P*=.01) times the odds of using dating apps than those who perceived *no risk*. Compared with those who perceived no risk, people who thought that the risk of having multiple sexual partners was *low* to *high* had 1.871 (95% CI 1.024-3.418; *P*=.04) times the odds of using dating apps. A significant number of app users (389/440, 88.4%) indicated that GNDAs should promote safe sex.

**Conclusions:**

This study identified that festival goers engaging in certain high-risk sexual behaviors, including casual sex, having multiple sexual partners, and having sex without discussion about STI status and boundaries, are more likely to use dating apps. Festival goers who perceived sex without any form of contraception, having sex while drunk, and having multiple sexual partners as risky were more likely to be app users. Policy makers and GNDA developers should acknowledge the vulnerability of their users to adverse sexual health outcomes and use GNDAs as a platform to promote risk-reduction practices.

## Introduction

### Background

Geosocial networking dating apps (GNDAs) provide users with a web-based platform to make social, romantic, or sexual connections. These apps connect users to potential partners based on their location. Such platforms have become increasingly popular since the launch of Tinder in 2012, which has grown to over 10 million users per day, with similar apps (Bumble and OkCupid) joining the market [1]. In Australia, web-based dating has become the second most preferred way to meet new partners after introductions from family and friends, surpassing more traditional methods of meeting [2]. A Dutch study on the primary motivations for Tinder use showed that in addition to dating, GNDAs may be used to ease communication and obtain self-worth or validation and for excitement or trendiness [1].

Young adults, the primary users of GNDAs [3], have a higher tendency toward risky sexual behaviors. For example, they often lack safe-sex discussions before intercourse [4], a significant risk factor for sexually transmitted infections (STIs). Consequently, in Australia, young adults (15-24 years) account for 50% of newly acquired STIs [5].

GNDA use has been directly associated with risky sexual behaviors. A meta-analysis concluded that GNDA users were more likely to contract STIs [6]. Similarly, Shapiro et al [7] found that Tinder users were more likely to report 5 or more previous sexual partners and a positive STI screen. This is concerning, given the high rate of GNDA use and promotion at music festivals [8]. Furthermore, a recent review of 99 studies on GNDAs and sexual health identified that the most common theme was risky sexual behaviors [9] and tended to focus on the lesbian, gay, bisexual, transgender, and queer (LGBTQ+) community [9].

Music festival attendees have also demonstrated significant risky sexual behaviors. An Australian study found that half of the music festival attendees reported taking illicit drugs in the last 12 months and had engaged in risky sexual behaviors such as sex without condoms, casual sex, and a lack of STI screening [10]. The environment of a music festival is particularly risky for dating app use because of the proximity of hundreds of young people, many of whom use dating apps and do not practice safe sex. Therefore, the proximity of congregated youth creates an environment conducive for increased app use and, in turn, greater potential for risky sexual behavior. There is evidence to suggest that GNDAs can be used for safe-sex health promotion, and several reviews have identified the potential effectiveness of mobile phone (or app based) interventions on delivering safe-sex information among young people [11,12]. Despite this potential, a 2016 study investigated 60 dating apps to determine whether they included any sexual health content. Huang et al [13] found that only 9 dating apps had sexual health content and 7 of these only targeted men having sex with men.

### Objective

In summary, GNDA use is prevalent among young adults, and festival goers are a higher risk group, but the association between sexual health and dating app use among young people attending festivals has, to our knowledge, not been investigated yet. Therefore, this study aims to explore GNDA use and risky sexual behavior among young adults attending a music festival.

## Methods

### Study Design

This study was a cross-sectional survey. People aged 18 years and above attending a music festival in 2019 in New South Wales, Australia, were eligible to participate in the study.

### Data Collection

Data were collected at a major 3-day music festival in 2019 in New South Wales, Australia. Recruitment was conducted by 5 of the authors who were on-site during the festival. Attendees who approached a permanent health promotion stall within the campgrounds were invited to take part and people within the target demographic were approached. Those approached were screened by age and given a participant information survey. The information sheet outlined the study aims, what they would be asked to do, the benefits of taking part and potential downsides, how the study was paid for, data storage, an explanation about study withdrawal, and how to contact the ethics committee or researchers. If willing to participate, paper surveys were distributed, completed anonymously, and placed in closed boxes for confidentiality. Survey completion was obtained as consent. Owing to the size, timing, and location of the festival, the data were collected via convenience sampling. The number of individuals who declined to participate was not recorded. No visibly intoxicated people were allowed to complete the study, and no incentives were provided. The data were entered into a Microsoft Excel spreadsheet.

### Survey Development

The questionnaire development was guided by public health and sexual health experts and included questions about demographics, sexual behavior, risk perception, and dating apps. Risky sexual behaviors were selected from the Safer Sex Behavior Questionnaire (SSBQ) by Dorio et al [14] and were used to develop questions in relation to dating apps and sexual health. Akin to the SSBQ, the questionnaire covered condom use, impact of intravenous drug use history in a sexual partner, drinking alcohol before sex, and discussion about safe sex with a partner before sexual activity. The SSBQ covered more risky sexual behaviors, such as engagement in anal intercourse and sexual intercourse on the first date. However, these were excluded to reduce survey fatigue, as it was necessary to keep the survey relatively brief. The survey was piloted (n=13) with university students, representing the target population and refined before being approved by the Western Sydney University Human Research Ethics Committee (H11327). The survey questions used in this study are listed in Multimedia Appendix 1.

### Outcome Measures

GNDA use and nonuse were determined by asking whether they had used a dating app in the last 12 months. Dating app users were also asked: “Should dating apps share educational messages about safe sex?”

The survey asked about reasons for use and sexual behavior with partners meeting off GNDAs. However, data were not captured in this study as it did not allow the comparison of users versus nonusers.

All participants were asked about their age, gender, sexual orientation, relationship, whether they had engaged in sexual activity in the last 12 months, and the number of sexual partners in the last 12 months. The survey explained the definition of *sexual activity* to be defined as oral and/or penetrative sex.

Participants were also asked what forms of contraception they used regularly, including *none*; *condoms*; *Plan B (morning-after pill)*; *the pill*; *intrauterine devices* *(IUDs), such as Mirena and Paragard*; *spermicide*; *Implanon—the rod;* *pulling out method*; or other methods. These were then categorized as follows for further analyses: *no contraception at all*, *condom use*, *Plan B*, *pulling out,* and *hormonal contraception*, with the latter including *the pill*, *IUD (Mirena and Paragard)*, *spermicide,* and *Implanon—the rod*.

With respect to sexual health behavior, participants were first asked about their actual behavior and then about their risk perception of this behavior. Participants were asked if they had engaged in the following 8 behaviors in the last 12 months: sex without any form of contraception; sex without condom but with other contraception, for example, the pill; casual sex; having sex while drunk; lack of discussion about STI status and sexual boundaries before sexual activity; having multiple sexual partners; having unprotected sex with a partner who has ever injected drugs; and finally having sex with a partner who has an STI. For each behavior, participants were asked to rate their perception of sexual risk on a 5-point Likert scale, ranging from no risk to very high risk.

### Data Analysis

Data were analyzed using SPSS, version 25. Bivariate analysis was used to compare categorical demographics, contraception, specific risky sexual behaviors, and related risk perceptions with dating app use. Statistical significance was defined as *P*=.05. Logistic regression analyses were used to estimate crude odds ratios (ORs) to determine the factors associated with using GNDAs. Contraceptive and specific sexual behavioral variables that were statistically significantly associated with GNDA use in bivariate analyses were then analyzed in a multivariate model, which was adjusted for sexual orientation, relationship status, and number of sexual partners.

## Results

### Demographics

About half (448/862, 51.9%) of the participants had used GNDAs within the past 12 months. A significant number of dating app users (389/440, 88.4%) indicated that GNDAs should promote safe sex. The study (N=862) was predominantly completed by those between the ages of 21 and 24 years (395/862, 45.8%), closely followed by those aged between 18 and 21 years (383/862, 44.4%; Table 1). The highest proportion of respondents were female (558/860, 64.9%), heterosexual (770/862, 89.3%), and sexually active (813/862, 94.3%). The majority were in exclusive relationships (362/858, 42.2%), whereas the rest were single and not dating (310/858, 36.1%) or casually dating or in open relationships (186/858, 21.7%). Of the sexually active participants (n=812), most had 2 to 5 sexual partners within the past 12 months (341/812, 41.9%), followed by those who had one sexual partner (311/812, 38.3%).

People identified as LGBTQ+ had almost twice the odds (OR 1.846, 95% CI 1.175-2.900; *P*=.008) of using GNDAs as compared with their heterosexual counterparts. Participants in exclusive relationships had a lower proportion of app usage (109/362, 30.1%) compared with casual daters (141/186, 75.8%) and singles (195/310, 62.9%). Casual daters were 7.273 times (95% CI 4.857-10.891; *P*<.001) more likely to have used GNDAs within 12 months compared with those in exclusive relationships. Participants who were *single and not dating* were 3.936 times (95% CI 2.853-5.430; *P*=.003) more likely to use GNDAs.

People who had more sexual partners were more likely to be dating app users. Of the participants with 1 partner, only 21.9% (68/311) were GNDA users, compared with 64.8% (221/341) of those with 2 to 5 partners and 80% (128/160) of those with more than 5 partners. Compared with those with only 1 partner in the last 12 months, participants with 2 to 5 partners had 6.581 times the odds (95% CI 4.643-9.328; *P*<.001) of using dating apps, whereas people with more than 5 partners had 14 times the odds of using dating apps (OR 14.294, 95% CI 8.92-22.906; *P*<.001).

**Table 1:** Logistic regression of geosocial networking dating app users and demographics (N=862).

Characteristics					Total, n (%)			Users, n (%)			Nonusers, n (%)			Dating app users, crude odds ratio (95% CI)			*P* value
**Age (years) (N=862)**																	
	18-20			383 (44.4)			192 (50.1)			191 (49.9)			1.054 (0.657-1.691)			.83	
	21-24			395 (45.8)			215 (54.4)			180 (45.6)			1.253 (0.782-2.007)			.35	
	25-30			84 (9.7)			41 (48.8)			43 (51.2)			Ref^a^			N/A^b^	
**Gender (n=860)**																	
	Female			558 (64.9)			291 (52.2)			267 (47.8)			Ref			N/A	
	Male			302 (35.1)			156 (51.7)			146 (48.3)			1.020 (0.771-1.350)			.89	
**Sexual orientation (N=862)**																	
	Heterosexual			770 (89.3)			388 (50.4)			382 (49.6)			Ref			N/A	
	**Nonheterosexual**													1.846 (1.175-2.900)			.008
		Homosexual	25 (2.9)			19 (76.0)			6 (24.0)								
		Bisexual	54 (6.3)			32 (59.3)			22 (40.7)								
		Other	13 (1.5)			9 (69.2)			4 (30.8)								
**Relationship status (n=858)**																	
	Single, not dating			310 (36.1)			195 (62.9)			115 (37.1)			3.936 (2.853-5.430)			.003	
	Casual dating or open relationship			186 (21.7)			141 (75.8)			45 (24.2)			7.237 (4.857-10.891)			<.001	
	Exclusive			362 (42.2)			109 (30.1)			253 (69.9)			Ref			N/A	
**Sexually active (N=862)**																	
	Yes			813 (94.3)			417 (51.3)			396 (48.7)			Ref			N/A	
	No			49 (5.7)			31 (63.3)			18 (36.7)			1.635 (0.900-2.907)			.11	
**Sexual partners in the past 12 months (n=812)**																	
	1			311 (38.3)			68 (21.9)			243 (78.1)			Ref			N/A	
	2-5			341 (42.0)			221 (64.8)			120 (35.2)			6.581 (4.643-9.328)			<.001	
	5+			160 (19.7)			128 (80.0)			32 (20.0)			14.294 (8.92-22.906)			<.001	

^a^Ref: reference (this is the comparison group used to establish the odds ratio).

^b^N/A: not applicable.

### Risky Sexual Behavior and User Status

#### Contraception

Table 2 shows that participants who used condoms had 1.914 times (95% CI 1.448-2.531; *P*<.001) the odds of using GNDAs. Participants who relied on emergency contraception as Plan B were more likely to use dating apps (OR 2.357, 95% CI 1.381-4.087; *P*=.002), whereas those who used hormonal contraception such as IUDs, the oral contraceptive pill, and the rod were less likely to use dating apps (OR 0.660, 95% CI 0.496-0.879; *P*=.004).

Table 3 shows that after adjusting for sexual orientation, relationship status, and number of sexual partners, no association was found between any form of contraception use and dating app use.

**Table 2:** Logistic regression of geosocial networking dating app users and sexual behaviors.

Behavior		Total, n (%)	Users, n (%)	Nonusers, n (%)	Dating app users, crude odds ratio (95% CI)	*P* value
**Contraception (n=811)**						
	No contraception at all	76 (15.7)	44 (57.9)	32 (43.3)	1.334 (0.828-2.162)	.24
	Condom use	417 (51.4)	247 (59.2)	170 (40.8)	1.914 (1.448-2.531)	<.001
	Plan B	67 (8.3)	47 (70.1)	20 (29.9)	2.357 (1.381-4.087)	.002
	Pulling out	211 (26)	114 (54)	97 (46)	1.152 (0.841-1.578)	.38
	Hormonal	505 (62.3)	240 (47.5)	265 (52.5)	0.660 (0.496-0.879)	.004
**Specific sexual behaviors (n=788)**						
	Sex without any form of contraception	385 (48.9)	205 (53.2)	180 (46.8)	1.227 (0.928-1.623)	.15
	Sex without a condom but with other contraception	524 (66.5)	262 (50)	262 (50)	0.927 (0.690-1.246)	.62
	Casual sex	413 (52.4)	280 (67.8)	133 (32.8)	4.567 (3.383-6.167)	<.001
	Sex while drunk	581 (73.7)	300 (51.6)	281 (48.4)	1.165 (0.848-1.600)	.35
	No discussion about STI^a^ status and/or boundaries before sex	278 (35.3)	189 (70)	89 (30)	3.024 (2.223-4.113)	<.001
	Multiple sexual partners	292 (37.1)	221 (75.7)	71 (24.3)	5.592 (4.043-7.736)	<.001
	Having unprotected sex with a PWID^b^	60 (7.6)	38 (63.3)	22 (36.7)	1.756 (1.018-3.028)	.04
	Having sex with a partner with an STI	52 (6.6)	35 (67.3)	17 (32.7)	2.110 (1.161-3.835)	.01

^a^STI: sexually transmitted infection.

^b^PWID: person who injects drugs.

**Table 3:** Multivariate logistic regression models of geosocial networking dating app use and sexual behaviors.

Characteristics			Dating app users, adjusted odds ratio (95% CI)		*P* value		Coefficient of determination (R^2^)		Chi-square (*df*)
**Contraception (n=811)**									
	Condom use^a^	1.334 (0.958-1.859)		.09		0.341		2.899 (1)	
	Plan B^a^	1.276 (0.698-2.331)		.42		0.338		0.639 (1)	
	Hormonal^a^	0.844 (0.601-1.186)		.33		0.338		0.955 (1)	
**Specific sexual behaviors (n=788)**									
	Casual sex^b^	3.096 (2.225-4.307)		<.001		0.272		45.346 (1)	
	No discussion about STI^c^ status and/or boundaries before sex^a^	1.755 (1.232-2.500)		.002		0.357		9.705 (1)	
	Multiple sexual partners^b^	3.943 (2.782-5.588)		<.001		0.295		62.261 (1)	
	Having unprotected sex with an PWID^a,d^	1.247 (0.649-2.399)		.51		0.345		0.444 (1)	
	Having sex with a partner with an STI^a^	1.645 (0.815-3.319)		.16		0.347		1.988 (1)	

^a^Adjusted for sexual orientation, relationship status, and number of sexual partners.

^b^Adjusted for sexual orientation and relationship status.

^c^STI: sexually transmitted infection.

^d^PWID: person who injects drugs.

#### Specific Sexual Behaviors

Table 2 shows that participants who had casual sex (*P*<.001), those who had no discussion about STIs or boundaries before having sex (*P*<.001), those with multiple sexual partners (*P*<.001), those having unprotected sex with a person who injects drugs (*P*=.04), and those having sex with a partner with an STI (*P*=.01) had higher odds of using GNDAs. Table 3 shows that after adjusting for sexual orientation and relationship status, those having casual sex have 3.096 times (95% CI 2.225-4.307; *P*<.001) the odds of using dating apps and those having multiple sexual partners have 3.943 times (95% CI 2.782-5.588; *P*<.001) the odds of using dating apps compared with those who have not. Similarly, after adjusting for sexual orientation, relationship status, and number of sexual partners, people who had no discussions before having sex about STIs or boundaries were more likely to use dating apps (OR 1.755, 95% CI 1.232-2.500; *P*=.002). However, no association was found between dating app usage and having unprotected sex with a person who injects drugs or having sex with a person with an STI, after adjusting for sexual orientation, relationship status, and number of sexual partners (Table 3).

#### Risk Perception of Risky Sexual Behavior and User Status

Table 4 shows that those who perceived the risk of having sex without any form of contraception to be *very high* had 2.486 times (95% CI 2.213-5.096; *P*=.01) the odds of using dating apps than those who saw *no risk*.

Those who felt that the risk of having sex while drunk to be *low to high* had 1.659 times (95% CI 1.067-2.581; *P*=.03) the odds of using dating apps compared with those who saw *no risk*. Similarly, those who felt the risk of having sex when drunk was *very high* were 2.151 times (95% CI 1.087-4.256; *P*=.03) more likely to use dating apps compared with those who felt there was no risk. Finally, people who thought that the risk of having multiple sexual partners was *low to high* had 1.871 times (95% CI 1.024-3.418; *P*=.04) the odds of using dating apps compared with people who thought having multiple partners posed *no risk*.

**Table 4:** Logistic regression of geosocial networking dating app users and the perceptions of risky sexual behavior.

Behavior and risk perception			Total, n (%)		Users, n (%)		Nonusers, n (%)		Dating app users, crude odds ratio (95% CI)		*P* value
**Sex without any form of contraception (n=628)**											
	No risk	40 (6.4)		15 (37.5)		25 (62.5)		Ref^a^		N/A^b^	
	Low to high risk	436 (69.4)		214 (49.1)		222 (50.9)		1.607 (0.825-3.131)		.16	
	Very high risk	152 (24.2)		91 (59.9)		61 (40.1)		2.486 (2.213-5.096)		.01	
**Sex without condom but with other contraception (n=663)**											
	No risk	69 (10.4)		28 (40.6)		41(59.4)		Ref		N/A	
	Low to high risk	561 (84.6)		288 (51.3)		273 (48.7)		1.545 (0.929-2.568)		.09	
	Very high risk	33 (5)		17 (51.5)		16 (48.5)		1.556 (0.675-3.585)		.30	
**Casual sex (n=610)**											
	No risk	94 (15.4)		52 (55.3)		42 (43.7)		Ref		N/A	
	Low to high risk	484 (79.3)		265 (54.8)		219 (45.2)		0.977 (0.627-1.524)		.92	
	Very high risk	32 (5.2)		20 (62.5)		12 (37.5)		1.346 (0.591-3.066)		.48	
**Sex while drunk (n=700)**											
	No risk	96 (13.7)		38 (39.6)		58 (60.4)		Ref		N/A	
	Low to high risk	551 (78.7)		287 (52.1)		264 (47.9)		1.659 (1.067-2.581)		.03	
	Very high risk	53 (7.6)		31 (58.5)		22 (41.5)		2.151 (1.087-4.256)		.03	
**No discussion about STIs^c^ and/or boundaries before sex (n=580)**											
	No risk	40 (6.9)		18 (45)		22 (55)		Ref		N/A	
	Low to high risk	370 (63.8)		196 (53)		174 (47)		1.377 (0.715-2.652)		.34	
	Very high risk	170 (29.3)		98 (57.6)		72 (42.4)		1.664 (0.832-3.327)		.15	
**Multiple sexual partners (n=581)**											
	No risk	48 (8.3)		20 (41.7)		28 (58.3)		Ref		N/A	
	Low to high risk	465 (80.3)		266 (57.2)		199 (42.8)		1.871 (1.024-3.418)		.04	
	Very high risk	68 (11.7)		37 (54.4)		31 (45.6)		1.671 (0.792-3.524)		.18	
**Having unprotected sex with an IVDU^d^ (n=497)**											
	No risk	46 (9.3)		22 (47.8)		24 (52.2)		Ref		N/A	
	Low to high risk	212 (42.7)		102 (48.1)		110 (51.9)		1.012 (0.534-1.915)		.97	
	Very high risk	239 (48.1)		131 (54.8)		108 (45.2)		1.323 (0.703-2.490)		.39	
**Having sex with a partner with an STI (n=493)**											
	No risk	42 (8.5)		21 (50)		21 (50)		Ref		N/A	
	Low to high risk	93 (18.9)		47 (50.5)		46 (49.5)		1.022 (0.493-2.118)		.95	
	Very high risk	358 (72.6)		191 (53.4)		167 (46.6)		1.144 (0.603-2.168)		.68	

^a^Ref: reference (this is the comparison group used to establish the odds ratio).

^b^N/A: not applicable.

^c^STI: sexually transmitted infection.

^d^IVDU: intravenous drug user.

## Discussion

### Principal Findings

This study explored the link between risky sexual behaviors, risk perceptions, and GNDA use among festival goers. After adjusting for confounders, statistically significant associations existed between GNDA use and lack of discussion about safe sex, engaging in casual sex, and having multiple sexual partners. Festival goers who perceived sex without any form of contraception, having sex while drunk, and having multiple sexual partners as risky were more likely to be app users. A high proportion of dating app users (389/440, 88.4%) also thought that GNDAs should promote safe sex.

Condom users were more likely to use dating apps, which can be seen as a protective factor; however, this was not significant after adjusting for confounders. People having unprotected sex with a person who injects drugs and having sex with a partner with an STI were about twice as likely to use dating apps. After adjusting for confounders, this effect was no longer apparent; however, this may have been because of the relatively small number of people in these categories. Given the prevalence of risky sexual behavior in this music festival population, it would be appropriate for GNDA companies and public health experts to include targeted health promotion interventions on such platforms [1,9,15].

Most participants were heterosexual (770/862, 89.3%), directly reflective of the general Australian population, where 11% identify as LGBTQ+ [16]. The LGBTQ+ group were almost twice as likely to use dating apps, a rate comparable with recent literature [3,4,9]. Rosenfeld et al [17] stated that over 60% of same-sex couples met on the web in 2008 and 2009 in the United States, positing a *thin dating market* for LGBTQ+ individuals. It is not surprising that those who are in an exclusive relationship are less likely to use dating apps, whereas those with multiple sexual partners are much more likely to use dating apps.

This study found that music festival attendees who engage in casual sex, do not discuss safe sex, have multiple partners, have unprotected sex with intravenous drug users, or someone with an STI are more likely to use dating apps. Peter and Valkenburg [18] had a similar finding that individuals who scored highly on *sexual permissiveness*—having more tolerant attitudes and higher engagement in risky sexual behaviors—were more likely to use GNDAs. Chan [19] also demonstrated that GNDA users have more casual relationships and sexual partners.

This high rate of casual sex in GNDA users attending music festivals is significant because of the increased STI risk [20]. In Australia, the rates of notifiable STIs such as gonorrhea and chlamydia have risen significantly in recent years [21], implying that there is a risk involved with having multiple sexual partners. In this study, people with multiple sexual partners were more likely to use dating apps. However, the effective use of barrier protection greatly diminishes the likelihood of STI contraction, with the male condom offering 90% protection against gonorrhea [22]. Those who perceived the risk of sex with no form of contraception to be *very high* were 2.5 times more likely to use dating apps and those using condoms were almost twice as likely to use dating apps, potentially signaling that dating app users are aware of the risks involved. A 2016 study found that young Australians have high rates of condom failures, with 48% experiencing the condom slipping off during intercourse and because of high rates of inconsistent or incorrect use [23]. In addition, the cost of condoms impedes their use, even in developed countries [24]. Therefore, a practical method to minimize STI contraction could be to promote awareness about correct use as opposed to increasing risk perception and the services that offer free condoms such as Aboriginal medical services and Family Planning New South Wales [25].

Furthermore, despite perceiving no contraception use to be at a *very high* risk, GNDA users at the festival had low rates of hormonal contraception and relied on emergency contraception. MacPhail et al [26] found that despite 75% of Australian university students having positive attitudes toward condom use, only 50% used condoms during their last encounter. Thus, an increasing perception of risk may not always translate to safer sexual behaviors. Given that Australia’s abortion rates are among the highest in the developed world [27], it is important to have greater health promotion on practical methods of reducing unintentional pregnancies. Those who did not use efficacious hormonal contraceptive options such as the oral contraceptive pill and IUDs [28] had higher odds of using GNDAs. Despite the high uptake of barrier contraception, its efficacy tends to be user dependent, as mentioned earlier [23]. Distributing information encouraging longer-acting contraceptive devices and correct contraceptive use via dating apps may be an effective means of curbing accidental pregnancies [28,29]. In addition, this study found that 37.1% (292/788) of sexually active respondents had multiple sexual partners in the last 12 months and that 48.9% (385/788) reported having not used any form of contraception. This is comparable with the study by Lim et al [30], which was conducted at the Big Day Out festival in 2007, with 48% of respondents reporting multiple partners in the last 3 months and 43% not using a condom because of substance abuse. Thus, it seems that over the past 13 years, no significant reduction in engagement in risky sexual behaviors among Australian music festival populations can be noted, and it remains an area in need of targeted sexual health promotion strategies.

### Limitations and Strengths

Study limitations included the use of a binary measure of dating app usage in the last 12 months, self-report, selection bias, and the inability to show causal effects because of the cross-sectional design. A longitudinal study method should be conducted to determine the relationship between GNDA use and sexual health behaviors and outcomes over time. Although the survey used evidence-based, risky sexual behaviors [14], the SSBQ was abridged to ensure respondents remained engaged and avoid survey fatigue. This may be problematic, as the study may not have captured the full breadth of risky sexual behaviors within the target demographic. Respondents were willing to complete the survey because the topic is of interest to the study population; however, we did not record how many people declined to partake.

Another study limitation is the potential influence of relationship status and sexual orientation on dating app usage. Our study found that LGBTQ+ people are twice as likely as heterosexuals to use dating apps. This may influence the results about the performance of risky sexual behaviors, as it has been shown that people of sexual minorities, especially men who have sex with men, engage in risky sexual behaviors. Similarly, an increased number of sexual partners and not being in an exclusive relationship were predictors of GNDA use. However, this was adjusted for in multivariate analyses. It should also be noted that participants could have been under the influence of drugs or alcohol, the data were therefore collected early in the day, and people who were perceived to be under the influence were excluded. Finally, the terminologies *casual sex* and *multiple sexual partners* may be considered vague and interpreted differently between participants. Thus, the results from questions in which these terms were used need to be interpreted with caution.

### Practical Implications

An overwhelming number of festival attendees who were GNDA users (389/440, 88.4%) indicated that safe-sex messaging should be included in GNDAs. Our study also demonstrated that young festival attendees engaging in more high-risk sexual behaviors were more likely to be app users. Therefore, as GNDA use at music festivals is promoted by app companies [8], it is recommended to focus on safe-sex messaging. This provides a platform for health strategists to target at-risk demographics. However, health professionals should be aware that a 2018 Australian survey found that although 88.7% of those aged between 18 and 29 years noticed sexual health promotion, only 40.9% considered it relevant and only 32% felt an increase in knowledge [15]. Therefore, the success of safe-sex messaging relies on whether the material will be relevant to users and increase their knowledge of risky sexual behavior. However, as stated earlier, increasing the perception of risk may not always translate to safer sexual behaviors. Despite relatively high levels of risk perception, health promotion messages to lower risk among music festival patrons should be further explored. Areas of future research include examining which GNDAs have the riskiest user bases and which strategies are most effective for harm reduction in this population. Focus groups with festival participants could be conducted to help ensure that messaging is relevant and targeted.

Major GNDAs such as Tinder and Bumble run web-based promotional subsidiaries, including *SwipeLife* (Tinder) [31], *The Buzz* (Bumble) [32], and blogs that incorporate safe-sex articles. As stated previously, less than 19% of heterosexual app users saw safe-sex messages in dating apps [9], despite existing literature identifying the importance of web-based safe sexual health messages to users [9,15,33]. Safe-sex messages may therefore need to appear more prominently within the apps themselves. This is further confirmed by our study, in which an overwhelming majority of participants were in favor of in-app sexual health resources. The fact that Tinder has released coronavirus safety messages in March 2020 demonstrates their ability and willingness to use health promotion messages [31]. In addition, intervention research for safer dating app use has also emerged [34]. Finally, there is evidence that mobile phone interventions can be successful in delivering safe-sex messages [35,36] and strengthens the argument that safe-sex messaging could appear more prominently within dating apps. However, a recent systematic review on new digital media interventions for sexual health promotion among young people reported that it should be taken into account that the technology itself does not necessarily lead to success [11]. The authors suggest that interventions should use high-quality, evidence-based content that engages with young people. Formative research [37] among Swedish youth on the development of a mobile phone app to promote safe sex identified that the following features would engage youth and therefore useful for the app development: *Condom Obstacles and Solutions; Quiz; Games; Self-Refection; Challenges; Stories by Peers (stories from peers and information from a doctor); Condom Tips, Pep Talk, and Boosting; and Random Facts*. Further guidelines are available for complex messaging in health promotion when developing interactive eHealth apps [38].

### Conclusions

This study identified that festival goers engaging in certain high-risk sexual behaviors, including casual sex, having multiple sexual partners, and having sex without discussion about STI status and boundaries, are more likely to use dating apps. Festival attendees who perceived sex without any form of contraception, having sex while drunk, and having multiple sexual partners as risky were more likely to be app users. A high proportion of dating app users support the notion that GNDAs should promote safe sex. The results of this study contribute to the growing body of knowledge surrounding the changing landscape of dating, sexual behaviors, and health impacts in the era of GNDAs. Policy makers and GNDA developers should acknowledge the vulnerability of their users to adverse sexual health outcomes and use GNDAs as a platform to promote risk-reduction practices.

## Appendix

Multimedia Appendix 1 Survey questions.

## Abbreviations

GNDA: geosocial networking dating app
IUD: intrauterine device
LGBTQ+: lesbian, gay, bisexual, transgender, and queer
OR: odds ratio
SSBQ: Safer Sex Behavior Questionnaire
STI: sexually transmitted infection
