# Supplementary material for: Geosocial Networking Dating App Usage and Risky Sexual Behavior in Young Adults Attending a Music Festival: Cross-sectional Questionnaire Study
Source: J Med Internet Res. 2021 Apr 15;23(4):e21082. doi: 10.2196/21082 (PMC8085743; doi:10.2196/21082)
Supplement: Multimedia Appendix 1 [file jmir_v23i4e21082_app1.docx]

Q.1. Age

Q.2. Gender

- Male
- Female
- Other

Q.3. Sexual orientation

- Heterosexual
- Homosexual
- Bisexual
- Asexual
- Pansexual
- Other, please specify

Q.4. Relationship status

- Single and not dating
- Casual dating
- In an open relationship
- In an exclusive relationship
- Other, please specify

Q.5. Have you engaged in sexual activity in the last 12 months?

- Yes, continue to question 6
- No, continue to question 9

Q.6. How many partners have you had sex with in the past 12 months?

- 1
- 2-5
- 5-10
- 10-15
- 15-30
- 30-50
- More than 50

Q.7. What forms of contraception do you use regularly? Tick as many that apply

- None
- Condoms
- Plan B (morning after pill)
- The Pill
- IUD (Mirena, Paragard)
- Spermicide
- Implanon “The rod”
- Pulling out method
- Other, please specify:

Q.8. Please tick the box if you have engaged in any of the following activities in the past 12 months. On a scale of 1 to 5, how much of a risk do you think these behaviours pose to your health?

*Please fill out the last column even if you have not engaged in these activities in the last 12 months*

| Activity | I have done this in the past 12 months | On a scale of 1 to 5, how much of a risk do you think these behaviours pose to your health? | | | | |
| --- | --- | --- | --- | --- | --- | --- |
|  |  | No risk | Mild risk | Moderate risk | High risk | Very high risk |
| Sex without any form of contraception |  |  |  |  |  |  |
| Sex without condom but with other contraception e.g. the pill |  |  |  |  |  |  |
| Casual sex |  |  |  |  |  |  |
| Having sex while drunk |  |  |  |  |  |  |
| Lack of discussion about STI status and sexual boundaries prior to sexual activity |  |  |  |  |  |  |
| Having multiple sexual partners |  |  |  |  |  |  |
| Having unprotected sex with a partner who has ever injected drugs |  |  |  |  |  |  |
| Having sex with a partner who has an STI |  |  |  |  |  |  |

Q.9. Have you used a dating app in the last 12 months?

- Yes, continue to question 11
- No, continue to question 10

NOTE: The rest of the survey questions were not used in data analysis and are therefore not presented.
